# Association between the hemoglobin A1c/High-density lipoprotein cholesterol ratio and stroke incidence: a prospective nationwide cohort study in China

**DOI:** 10.1186/s12944-025-02438-4

**Published:** 2025-01-25

**Authors:** Chaojuan Huang, Hongtao You, Yuyang Zhang, Ligang Fan, Xingliang Feng, Naiyuan Shao

**Affiliations:** 1 Department of Neurology, The First Affiliated Hospital of Anhui Medical University, Hefei, Anhui 230022 China; 2 Department of Neurosurgery, The Third Affiliated Hospital of Soochow University, Changzhou, Jiangsu 213000 China; 3 Department of Neurosurgery, The First People’s Hospital of Changzhou, Changzhou, Jiangsu 213000 China; 4 Department of Urology, The First Affiliated Hospital of Anhui Medical University, Hefei, Anhui 230022 China; 5 Department of Urology, The Third Affiliated Hospital of Soochow University, Changzhou, Jiangsu 213000 China; 6 Department of Urology, The First People’s Hospital of Changzhou, Changzhou, Jiangsu 213000 China

**Keywords:** Hemoglobin A1c, High-Density Lipoprotein Cholesterol, HbA1c/HDL-C Ratio, Stroke, CHARLS

## Abstract

**Background:**

Stroke has emerged as an escalating public health challenge among middle-aged and older individuals in China, closely linked to glycolipid metabolic abnormalities. The Hemoglobin A1c/High-Density Lipoprotein Cholesterol (HbA1c/HDL-C) ratio, an integrated marker of glycolipid homeostasis, may serve as a novel predictor of stroke risk.

**Methods:**

Our investigation utilized data from the China Health and Retirement Longitudinal Study cohort (2011–2018). Stroke cases were identified based on self-reported, physician-confirmed diagnoses. Logistic regression models were established to determine the correlation between HbA1c/HDL-C and stroke prevalence (2011) as well as between cumulative mean HbA1c/HDL-C (2011–2015) and new stroke incidence (2015–2018). Additionally, smoothed curve fitting, subgroup analyses, and interaction tests were conducted to ensure the robustness of the findings.

**Results:**

In the cross-sectional analysis, 8,502 participants were enrolled, of whom 189 had a history of stroke. Our findings revealed a significant positive linear relationship between HbA1c/HDL-C and stroke prevalence after adjusting for covariates (OR: 1.26, 95% CI: 1.09–1.45). When HbA1c/HDL-C was categorized into tertiles, only the highest tertile (T3) showed a significant correlation with stroke prevalence compared to the lowest tertile (T1) (OR:1.71, 95% CI: 1.05–2.77). In the longitudinal analysis of 5,165 participants, 336 cases of new-onset stroke were identified over a follow-up period of 7 years. Adjusting for confounders, individuals with higher cumulative mean HbA1c/HDL-C exhibited an increased likelihood of new stroke incidence (OR: 1.14, 95% CI: 1.01–1.29). Using the T1 of cumulative mean HbA1c/HDL-C as a reference, the fully adjusted OR for stroke was 1.65 (95% CI: 1.21–2.24) in T2 and 1.54 (95% CI: 1.08–2.19) in T3. The predictive value of the HbA1c/HDL-C in stroke risk assessment have been significantly improved compared to the traditional HDL-C and HbA1c. Consistent associations were observed across most stratified subgroups.

**Conclusions:**

Elevated baseline and cumulative mean HbA1c/HDL-C levels are significantly associated with an increased risk of stroke among middle-aged and older individuals in China, underscoring the potential of HbA1c/HDL-C as a clinical marker for long-term stroke risk assessment and prevention strategies.

**Supplementary Information:**

The online version contains supplementary material available at 10.1186/s12944-025-02438-4.

## Introduction

Stroke is a medical emergency characterized by sudden neurological deficits [1]. As reported by the World Stroke Organization, it is the leading cause of disability and death worldwide [2], placing a substantial social and economic burden [3]. Over the past 30 years, the incidence, prevalence, and mortality of stroke have risen considerably, particularly in low- and middle-income countries such as South Africa and regions of Asia [4], with China bearing the highest lifetime risk and prevalence of stroke globally [5]. Epidemiological investigations in the Chinese population aged 40 years and older estimated that, in 2020, stroke incidence, prevalence, and mortality reached 3.4, 17.8, and 2.3 million cases, respectively [6]. Alarmingly, nearly 12.5% of stroke patients in China experienced recurrence within one year [7]. This rapidly growing prevalence and recurrence rate have heightened awareness within global health systems, underscoring the urgent need for effective prevention and control strategies. Identifying modifiable indicators for predicting stroke and enabling early intervention are crucial steps in addressing this challenge.

Stroke is primarily driven by metabolic factors such as hypertension, diabetes mellitus (DM), and dyslipidemia, as well as behavioral factors like smoking and poor dietary nutrient balance, and environmental influences including air pollution and low socioeconomic status [4, 8, 9]. Among these, metabolic-related risks—specifically, abnormal glucose and lipid metabolism—contribute to 21.9% and 21.6% to the stroke burden, respectively [4]. Hemoglobin A1c (HbA1c) reflects average glucose metabolism over the past 2–3 months [10]. High-density lipoprotein cholesterol (HDL-C), often referred to as “good” cholesterol, assists in reverse cholesterol transport from peripheral tissues to the liver, exerting protective effects on blood vessels, and offering anti-inflammatory and antioxidant properties [11]. A recent study involving 11,220 participants over a 7.5-year follow-up demonstrated that each 1-unit increase in baseline and long-term HbA1c corresponded to a 10% and 12% increase in stroke risk, respectively [12]. Regarding HDL-C, a cross-sectional study indicated an inverse association with stroke risk for levels below 1.55 mmol/L [13]. Additionally, longitudinal research by Ali et al. reported that low HDL-C levels were independently associated with a 2.24-fold increase in 1-year stroke recurrence risk [14]. Mendelian randomization studies further support the causal roles of HbA1c and HDL-C in stroke risk [15, 16]. Accumulating evidence suggests complex interactions between glucose and lipid metabolism [17]. A large retrospective study revealed that the connection between low HDL-C and stroke risk progressively intensified with worsening glycemic status (from normal to prediabetes and DM) [18]. Higher HDL-C levels may improve glucose metabolism through strengthening β-cell function, increasing insulin secretion and sensitivity, and enhancing glucose absorption and utilization [19, 20]. Conversely, elevated glucose levels can inhibit lipid breakdown, accelerate free fatty acid synthesis, and promote cholesterol accumulation, thereby disrupting lipid homeostasis and creating a vicious cycle [21]. Accordingly, estimating the impact of HbA1c or HDL-C alone on stroke risk may be insufficient. Few studies to date have examined the association between combined glycolipid metabolic indicators and stroke risk.

The HbA1c/HDL-C index was first proposed by Hu et al. in 2021 [22] as a comprehensive indicator of glycolipid metabolism. This easily attainable index has since been applied to evaluate its relevance to carotid atherosclerosis [22], diabetic retinopathy [23], metabolic disease [21], and other conditions. Despite its potential clinical significance, no studies to date have examined the association between the HbA1c/HDL-C index and stroke risk. This study intends to systematically evaluate the association between HbA1c/HDL-C and stroke prevalence, as well as the relationship between cumulative mean HbA1c/HDL-C and new stroke incidence. The publicly available China Health and Retirement Longitudinal Study (CHARLS) cohort of the Chinese population provides the foundation for this investigation. We hypothesized that (i) HbA1c/HDL-C is significant and positively associated with stroke prevalence in the cross-sectional analysis, and (ii) cumulative mean HbA1c/HDL-C exerts a consistent and detrimental impact on new stroke incidence in the longitudinal analysis.

## Methods

### Study design and population

The data for our research were derived from the CHARLS cohort, a large-scale, prospective, nationwide cohort that includes individuals aged 45 years and older [24]. CHARLS achieved national representativeness through a multistage, stratified probability sampling design, which randomly selected participants from 450 communities across 150 counties in 28 provinces in China. Baseline data were collected from June 2011 to March 2012 (Wave 1) by trained staff through face-to-face interviews. Sociodemographic factors and health status were assessed biennially using a standardized questionnaire. Initially, 17,708 participants were enrolled in Wave 1, and follow-up was conducted every two years, with Wave 2 in 2013, Wave 3 in 2015, and Wave 4 in 2018. Blood samples were collected at baseline (Wave 1) and again in 2015 (Wave 3).

**Fig. 1:**
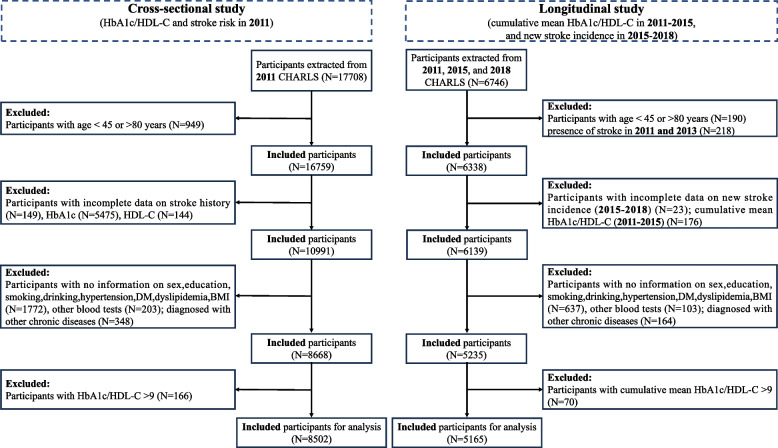
Flowchart of the population screening procedure. Abbreviations: HbA1c, hemoglobin A1c; HDL-C, high-density lipoprotein cholesterol; CHARLS, China Health and Retirement Longitudinal Study; DM, diabetes mellitus; BMI, body mass index

Participants aged 45 to 80 years with complete information on HbA1c, HDL-C, and stroke history were included in the study. The specific exclusion criteria for the cross-sectional analysis included: (1) incomplete information on stroke history in Wave 1; (2) missing data on HbA1c or HDL-C in Wave 1; (3) extreme values of HbA1c/HDL-C, defined as more than 3 standard deviations from the mean (> 9). The specific exclusion criteria for the longitudinal analysis included: (1) presence of stroke in Waves 1 and 2 or incomplete information on new stroke incidence in Waves 3 and 4. (2) missing data on HbA1c or HDL-C in Waves 1 and 2; (3) extreme values of cumulative mean HbA1c/HDL-C (> 9). The common exclusion criteria were as follows: (1) age outside the range of 45–80 years; (2) missing data on sex, education, smoking, drinking, body mass index (BMI), total cholesterol (TC), triglyceride (TG), HDL-C, low-density lipoprotein cholesterol (LDL-C), or fasting blood glucose (FBG); (3) missing information on comorbid conditions, including hypertension, DM, dyslipidemia, chronic lung disease, or heart disease; (4) a diagnosis of chronic diseases such as cancer, memory-related diseases, depression, or arthritis. A sum of 8,502 participants were included in the cross-sectional analysis, and 5,165 participants were included in the longitudinal analysis. Detailed inclusion and exclusion procedures are depicted in Fig. 1.

The publicly available CHARLS datasets can be assessed through the official website (http://charls.pku.edu.cn/). Ethical approval was granted by the Peking University Institutional Review Board (IRB00001052-11015), and signed informed consent was obtained from each subject prior to inclusion. The data were extracted for secondary analysis, thereby obviating the need for additional ethical approval.

### Assessment of exposure

Fasting venous blood samples were collected from all participants by trained medical staff after an overnight fast of at least 8 h to ensure the accuracy of metabolic measurements. The blood samples were then centrifuged, frozen at -20 °C, and shipped to the central laboratory in Beijing for analysis within two weeks. Upon arrival, the samples were stored in a deep freezer at -80 °C until assayed. HbA1c and HDL-C levels were measured using boronate affinity high-performance liquid chromatography and an enzymatic colorimetric assay, respectively. The exposure variable for the cross-sectional analysis was the HbA1c/HDL-C in 2011, while the exposure variable for the longitudinal analysis was the cumulative mean HbA1c/HDL-C. The cumulative mean HbA1c/HDL-C was calculated as: (HbA1c/HDL-C of 2011 + HbA1c/HDL-C of 2015) / 2. Additionally, the continuous exposure variable was categorized into tertiles for further analysis.

### Assessment of outcome

The outcome of the cross-sectional analysis was stroke risk in 2011 (history of stroke = 1, no history of stroke = 0). In the longitudinal analysis, participants were free of stroke in 2011 and 2013, and the outcome was new stroke incidence in 2015–2018 (new stroke = 1, no new stroke = 0). Stroke determination was identified through a self-reported standardized questionnaire, which asked participants: “Have you ever been diagnosed with a stroke by a doctor?” and “Have you been diagnosed with stroke by a doctor since the last follow up visit?” [25].

### Ascertainment of covariates

Our study enrolled demographic information, anthropometric parameters, behavioral features, health status, and laboratory test results as potential covariates that may influence the association between HbA1c/HDL-C and stroke. (1) Demographic information included age, sex, educational level, marital status, and residence. Age was stratified into < 60/ ≥ 60 years; sex was grouped as female/male; educational level was sorted into primary school or lower, middle school or higher [26]; marital status was classified as married/non-married; and residence was divided into rural/urban. (2) Anthropometric parameters included BMI, calculated as BMI (kg/m^2^) = weight (kg) / height^2^ (m^2^), and categorized as < 24/24–28/ ≥ 28. (3) Behavioral features included smoking and drinking behavior. Smoking behavior was classified as “No” for never smokers and “Yes” for former or current smokers [27]; drinking behavior was categorized as “No” or “Yes”, based on whether the participant had consumed alcohol in the past year [26]. (4) Health status encompassed hypertension, DM, dyslipidemia, chronic lung disease, and heart disease. Hypertension was determined according to established guidelines [28] as either systolic blood pressure ≥ 140 mmHg, diastolic blood pressure ≥ 90 mmHg, diagnosed by a physician, or use of antihypertensive therapy. DM was diagnosed based on FBG ≥ 7.0 mmol/L, random glucose ≥ 11.1 mmol/L, HbA1c ≥ 6.5%, diagnosed by a physician, or use of hypoglycemic medication [29]. Dyslipidemia was defined by TC ≥ 6.2 mmol/L, TG ≥ 2.3 mmol/L, HDL-C < 1.0 mmol/L, LDL-C ≥ 4.1 mmol/L, diagnosed by a physician, or use of lipid-lowering treatment [30]. Subjects were identified as having chronic lung disease or heart disease if they had been definitively diagnosed with these conditions by a physician. (5) Laboratory tests contained hemoglobin, TC, TG, FBG, blood urea nitrogen (BUN), creatinine, and uric acid (UA).

### Missing data handling

To evaluate potential biases due to non-responders, we have included a comparison of the baseline characteristics between responders and non-responders in both the cross-sectional (2011) and longitudinal (2011–2018) analyses, as shown in Supplementary Tables 1 and 2. The results indicate that responders in the cross-sectional analysis were more likely to be married females residing in rural areas, with lower educational levels, a higher prevalence of hypertension, DM, and dyslipidemia, as well as elevated lipid levels but lower glucose and HbA1c/HDL-C levels compared to non-responders. Furthermore, the distribution patterns of baseline characteristics between responders and non-responders were similar in the longitudinal analysis. Given the high proportion of missing data in the CHARLS dataset, we opted for complete case analysis in this study, including only participants with complete data for all variables of interest.

### Statistical analysis

In the cross-sectional analysis, we first conducted a statistical description of the baseline characteristics across groups. Continuous variables are expressed as mean ± standard deviation (SD) and compared employing two-sample t-test or analysis of variance. Categorical variables are exhibited as frequencies (proportions), with group differences assessed using chi-square tests. Univariable and multivariable logistic regression analyses were established to evaluate the independent relationships between HbA1c/HDL-C and stroke risk in 2011. Both continuous and tertile forms of the HbA1c/HDL-C variables were engaged in the regression models. The corresponding effect sizes are depicted as odds ratios (OR) with 95% confidence intervals (95% CI). Four models were applied: the crude model (unadjusted for any covariates), Model 1 (adjusted for age, sex, education, residence, marital status, and BMI), Model 2 (adjusted for smoking and drinking behavior built upon Model 1), and Model 3 (further adjusted for hypertension, DM, dyslipidemia, chronic lung disease, heart disease, TC, TG, FBG, UA, and hemoglobin based on Model 2). The covariates included in the regression model were selected based on their relevance to HbA1c/HDL-C or stroke, as identified in previous studies. All variables met the criteria for logistic regression analysis, with no significant multicollinearity observed (variance inflation factors for all variables were below 5) (Supplementary Table 3). Trend tests were performed using the median values of each HbA1c/HDL-C tertile.

Subsequently, a multivariable-adjusted restricted cubic spline (RCS) analysis combined with segmented regression were employed to intuitively visualize the dose–response relationship between HbA1c/HDL-C levels and stroke risk. We applied the Akaike Information Criterion (AIC) and Bayesian Information Criterion (BIC) to determine the optimal number of knots. Based on the lowest AIC and BIC values, the optimal number of knots was identified as 3 in the cross-sectional and longitudinal analysis. The presence of distinct inflection points in the graphics, along with a *p*-value for nonlinear analyses less than 0.05, indicated a nonlinear correlation. Additionally, we evaluated the discriminative performance of HbA1c, HDL-C, and the HbA1c/HDL-C by analyzing their receiver operating characteristic (ROC) curves and calculating the corresponding area under the curve (AUC). To further evaluate the incremental predictive value of the HbA1c/HDL-C, we compared it with HbA1c and HDL-C individually using the net reclassification index (NRI) and integrated discrimination improvement (IDI) metrics.

Furthermore, subgroup analyses were conducted to investigate the consistent influence of HbA1c/HDL-C (both continuous and categorical) on stroke risk across various subgroups. Stratified logistic regression models were performed for factors including age, sex, education, marital status, BMI, smoking behavior, drinking behavior, hypertension, DM, and dyslipidemia. Likelihood ratio tests were conducted to estimate potential interaction terms. Similar univariable and multivariable logistic regression analyses, along with RCS and subgroup analyses, were utilized to investigate the associations between cumulative mean HbA1c/HDL-C (2011–2015) and new stroke incidence in 2015–2018 within the longitudinal study. All statistical analyses were completed using EmpowerStats (http://www.empowerstats.com) and R software version 4.4.1 (http://www.R-project.org). A *p*-value < 0.05 was recognized as reaching statistical significance (two-sided).

## Results

### Characteristics of study population

Ultimately, 8,502 individuals were included in the cross-sectional analysis, with a mean age of 58.91 ± 8.74 years; among them, 4,540 (53.40%) were female. HbA1c/HDL-C levels were approximately normally distributed, with a mean (SD) of 4.25 (1.33) (Supplementary Fig. 1A). Detailed differences in clinical characteristics by HbA1c/HDL-C tertiles are described in Table 1. In the longitudinal study, 5,165 individuals were included, with a baseline age of 58.35 ± 8.24 years, of which 55.51% were female. After a mean follow-up of seven years, 336 participants experienced new stroke incidence. The cumulative mean HbA1c/HDL-C from 2011 to 2015 was 4.48 ± 1.18 for the entire group (Supplementary Fig. 1B). Baseline clinical characteristics grouped by cumulative mean HbA1c/HDL-C tertiles are outlined in Table 2. Compared to the first tertile (T1), individuals in the second and third tertiles (T2 and T3) were more likely to be male, married, urban residents, and to have higher educational attainment and BMI in both the cross-sectional and longitudinal studies (*p* < 0.05). Smoking and drinking behaviors also differed significantly among the groups (*p* < 0.05). Chronic diseases, such as hypertension, DM, dyslipidemia, and heart disease, gradually increased with rising HbA1c/HDL-C and cumulative mean HbA1c/HDL-C tertiles (*p* < 0.05). Regarding laboratory tests, higher tertile groups exhibited increased levels of hemoglobin, TG, FBG, creatinine, and UA but lower TC and BUN values compared to T1 (*p* < 0.05). Age and the prevalence of chronic lung disease did not differ significantly across groups. Furthermore, summary characteristics were compared between individuals with and without stroke (Supplementary Tables 4 and 5).

**Table 1:** Baseline population characteristics of the cross-sectional study across HbA1c/HDL-C tertiles in 2011

Characteristic	Total (*n* = 8502)	Tertiles			*P* value
		T1 (1.34,3.53) (*n* = 2828)	T2 (3.53,4.61) (*n* = 2840)	T3 (4.61,8.99) (*n* = 2834)	
Age, year	58.91 ± 8.74	59.27 ± 8.95	58.68 ± 8.69	58.77 ± 8.56	0.05
Age, n (%)					0.16
< 60	4712(55.42)	1530(53.99)	1602(56.41)	1580(55.87)	
> = 60	3790(44.58)	1304(46.01)	1238(43.59)	1248(44.13)	
Female, *n* (%)	4540(53.40)	1563(55.15)	1566(55.14)	1411(49.89)	** < 0.0001**
Education, *n* (%)					** < 0.001**
Primary school or lower	5973(70.25)	2063(72.79)	1997(70.32)	1913(67.64)	
Middle school or higher	2529(29.75)	771(27.21)	843(29.68)	915(32.36)	
Marital status, *n* (%)					** < 0.01**
Married	7556(88.87)	2477(87.40)	2546(89.65)	2533(89.57)	
Non-Married	946(11.13)	357(12.60)	294(10.35)	295(10.43)	
Residence, *n* (%)					** < 0.0001**
Rural area	5581(65.64)	2029(71.59)	1863(65.60)	1689(59.72)	
Urban	2921(34.36)	805(28.41)	977(34.40)	1139(40.28)	
BMI, kg/m^2^					** < 0.0001**
< 24	5822(68.48)	2349(82.89)	1971(69.40)	1502(53.11)	
24–28	2267(26.66)	432(15.24)	720(25.35)	1115(39.43)	
> = 28	413(4.86)	53(1.87)	149(5.25)	211(7.46)	
Smoking, *n* (%)	3348(39.38)	1100(38.81)	1059(37.29)	1189(42.04)	** < 0.001**
Drinking, *n* (%)	2797(32.90)	1100(38.81)	859(30.25)	838(29.63)	** < 0.0001**
Hypertension, *n* (%)	3450(40.58)	977(34.47)	1130(39.79)	1343(47.49)	** < 0.0001**
DM, *n* (%)	1152(13.55)	193(6.81)	275(9.68)	684(24.19)	** < 0.0001**
Dyslipidemia, *n* (%)	3380(39.76)	548(19.34)	732(25.77)	2100(74.26)	** < 0.0001**
Heart disease, *n* (%)	971(11.42)	254(8.96)	311(10.95)	406(14.36)	** < 0.0001**
Chronic lung disease, *n* (%)	900(10.59)	322(11.36)	311(10.95)	267(9.44)	0.05
Stroke, *n* (%)	189(2.22)	36(1.27)	52(1.83)	101(3.57)	** < 0.0001**
Hemoglobin, g/dL	14.39 ± 2.21	14.10 ± 2.10	14.38 ± 2.18	14.68 ± 2.31	** < 0.0001**
TC, mg/dL	193.75 ± 38.19	199.05 ± 35.91	192.12 ± 37.53	190.09 ± 40.43	** < 0.0001**
TG, mg/dL	128.15 ± 85.22	88.33 ± 40.34	117.90 ± 61.54	178.35 ± 110.41	** < 0.0001**
LDL-C, mg/dL	117.25 ± 34.58	116.78 ± 33.29	120.46 ± 33.65	116.78 ± 33.29	** < 0.01**
FBG, mg/dL	108.55 ± 31.30	102.19 ± 19.28	104.49 ± 23.89	119.01 ± 42.83	** < 0.0001**
BUN, mg/dL	15.70 ± 4.48	16.15 ± 4.67	15.61 ± 4.44	15.33 ± 4.29	** < 0.0001**
Creatinine, mg/dL	0.78 ± 0.20	0.77 ± 0.20	0.78 ± 0.19	0.80 ± 0.20	** < 0.0001**
UA, mg/dL	4.44 ± 1.25	4.29 ± 1.21	4.40 ± 1.21	4.64 ± 1.29	** < 0.0001**
HbA1c/HDL-C	4.25 ± 1.33	2.94 ± 0.42	4.04 ± 0.30	5.77 ± 0.97	** < 0.0001**

*HbA1c* Hemoglobin A1c, *HDL-C* High-density lipoprotein cholesterol, *BMI* Body mass index, *DM* Diabetes mellitus, *TC* Total cholesterol, *TG* Triglyceride, *LDL-C* Low-density lipoprotein cholesterol, *FBG* Fasting blood glucose, *BUN* Blood urea nitrogen, *UA* Uric acid

**Table 2:** Baseline population characteristics of the longitudinal study across HbA1c/HDL-C tertiles in 2011

Characteristic	Total (*n* = 5165)	Tertiles			*P* value
		T1 (1.65,3.88) (*n* = 1728)	T2 (3.88,4.81) (*n* = 1718)	T3 (4.81,8.97) (*n* = 1719)	
Age, year	58.35 ± 8.24	58.48 ± 8.31	58.13 ± 8.26	58.43 ± 8.13	0.93
Age, *n* (%)					0.65
< 60	2942(56.96)	972(56.25)	993(57.80)	977(56.84)	
> = 60	2223(43.04)	756(43.75)	725(42.20)	742(43.16)	
Female, *n* (%)	2867(55.51)	1012(58.56)	977(56.87)	878(51.08)	** < 0.0001**
Education, *n* (%)					** < 0.001**
Primary school or lower	3634(70.36)	1268(73.38)	1206(70.20)	1160(67.48)	
Middle school or higher	1531(29.64)	460(26.62)	512(29.80)	559(32.52)	
Marital status, *n* (%)					** < 0.01**
Married	4667(90.36)	1536(88.89)	1549(90.16)	1582(92.03)	
Non-Married	498(9.64)	192(11.11)	169(9.84)	137(7.97)	
Residence, *n* (%)					** < 0.0001**
Rural area	3509(67.94)	1259(72.86)	1166(67.87)	1084(63.06)	
Urban	1656(32.06)	469(27.14)	552(32.13)	635(36.94)	
BMI, kg/m^2^					** < 0.0001**
< 24	3004(58.16)	1300(75.23)	998(58.09)	706(41.07)	
24–28	1745(33.79)	363(21.01)	588(34.23)	794(46.19)	
> = 28	416(8.05)	65(3.76)	132(7.68)	219(12.74)	
Smoking, *n* (%)	1936(37.48)	610(35.30)	622(36.20)	704(40.95)	** < 0.01**
Drinking, *n* (%)	1692(32.76)	656(37.96)	532(30.97)	504(29.32)	** < 0.0001**
Hypertension, *n* (%)	1999(38.70)	569(32.93)	627(36.50)	803(46.71)	** < 0.0001**
DM, *n* (%)	656(12.70)	107(6.19)	131(7.63)	418(24.32)	** < 0.0001**
Dyslipidemia, *n* (%)	2077(40.21)	360(20.83)	503(29.28)	1214(70.62)	** < 0.0001**
Heart disease, *n* (%)	553(10.71)	145(8.39)	175(10.19)	233(13.55)	** < 0.0001**
Chronic lung disease, *n* (%)	513(9.93)	182(10.53)	169(9.84)	162(9.42)	0.55
New stroke incidence, *n* (%)	336(6.51)	75(4.34)	122(7.10)	139(8.09)	** < 0.0001**
Hemoglobin, g/dL	14.39 ± 2.17	14.10 ± 2.06	14.44 ± 2.17	14.62 ± 2.25	** < 0.0001**
TC, mg/dL	193.52 ± 38.01	198.80 ± 35.47	191.33 ± 37.14	190.41 ± 40.70	** < 0.0001**
TG, mg/dL	130.58 ± 93.65	92.63 ± 45.51	120.97 ± 65.31	178.33 ± 127.26	** < 0.0001**
LDL-C, mg/dL	116.71 ± 34.37	116.71 ± 32.74	119.31 ± 33.07	114.11 ± 36.96	**0.01**
FBG, mg/dL	108.19 ± 30.92	101.76 ± 17.71	104.18 ± 19.33	118.67 ± 44.92	** < 0.0001**
BUN, mg/dL	15.64 ± 4.32	16.07 ± 4.62	15.40 ± 4.12	15.45 ± 4.18	** < 0.0001**
Creatinine, mg/dL	0.77 ± 0.18	0.75 ± 0.18	0.76 ± 0.17	0.78 ± 0.19	** < 0.0001**
UA, mg/dL	4.36 ± 1.21	4.22 ± 1.16	4.30 ± 1.19	4.56 ± 1.26	** < 0.0001**
Cumulative mean HbA1c/HDL-C	4.48 ± 1.18	3.31 ± 0.41	4.32 ± 0.26	5.81 ± 0.89	** < 0.0001**

*HbA1c* Hemoglobin A1c, *HDL-C* High-density lipoprotein cholesterol, *BMI* Body mass index, *DM* Diabetes mellitus, *TC* Total cholesterol, *TG* Triglyceride, *LDL-C* Low-density lipoprotein cholesterol, *FBG* Fasting blood glucose, *BUN* Blood urea nitrogen, *UA* Uric acid

### Association between HbA1c/HDL-C and stroke risk at baseline

**Fig. 2:**
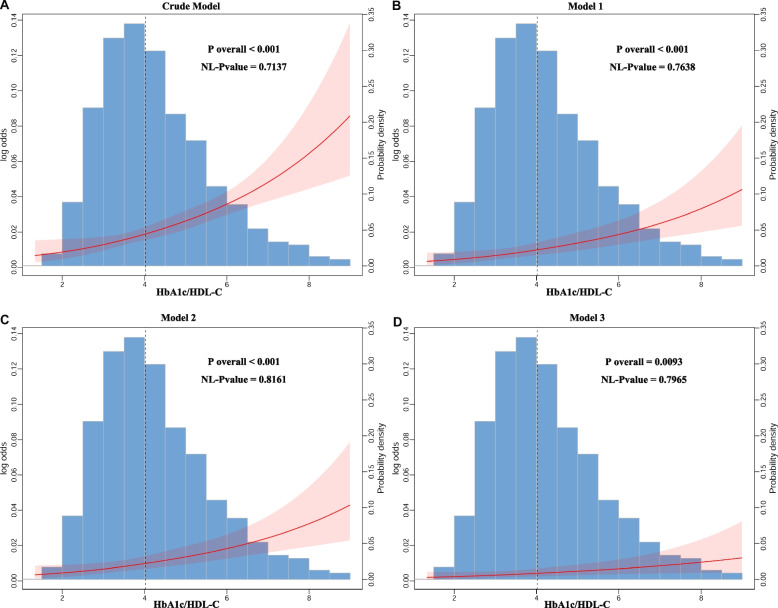
RCS curves are shown to reflect the dose–response association between HbA1c/HDL-C and stroke risk with different covariate adjustments. Odds ratios were represented by red solid lines, and 95% confidence intervals by red shaded areas. Abbreviations: RCS, Restricted cubic spline; HbA1c, hemoglobin A1c; HDL-C, high-density lipoprotein cholesterol

The results of logistic regression demonstrated a steady association between HbA1c/HDL-C levels and stroke risk in 2011 (Table 3). Continuous HbA1c/HDL-C levels were positively related to stroke risk after adjusting for various covariates. The effect sizes were as follows: 1.40 (95% CI: 1.27–1.53) in the crude model, 1.33 (95% CI: 1.21–1.48) in minimally adjusted Model 1, 1.32 (95% CI: 1.19–1.46) in partially adjusted Model 2, and 1.26 (95% CI: 1.09, 1.45) in fully adjusted Model 3. Consistently, when continuous HbA1c/HDL-C was converted to a categorical variable based on tertiles, a positive relationship between HbA1c/HDL-C tertiles and stroke risk was observed. Using the T1 of HbA1c/HDL-C as the reference, elevated levels were associated with a 27% increase in stroke risk for T2 (adjusted OR = 1.27, 95% CI: 0.81–1.98, *p* = 0.29) and a 71% increase for T3 (adjusted OR = 1.71, 95% CI: 1.05–2.77, *p* = 0.03). A statistically significant trend was observed between HbA1c/HDL-C tertiles and stroke risk (*p* for trend < 0.05). As presented in Fig. 2, the smooth curve analysis demonstrated a positive linear correlation between HbA1c/HDL-C and stroke risk across all models (*p* for nonlinearity > 0.05).

**Table 3:** The cross-sectional study that exploring the associations between HbA1c/HDL-C and stroke prevalence in 2011

HbA1c/HDL-C	Stroke	
	OR (95% CI)	*P* value
Crude Model		
Continuous	1.40(1.27,1.53)	< 0.0001
Categories		
Tertile1	ref	ref
Tertile2	1.45(0.94,2.22)	0.09
Tertile3	2.88(1.96,4.23)	< 0.0001
*P* for trend		< 0.0001
Model 1		
Continuous	1.33(1.21,1.48)	< 0.0001
Categories		
Tertile1	ref	ref
Tertile2	1.37(0.89,2.12)	0.15
Tertile3	2.45(1.63,3.66)	< 0.0001
*P* for trend		< 0.0001
Model 2		
Continuous	1.32(1.19,1.46)	< 0.0001
Categories		
Tertile1	ref	ref
Tertile2	1.35(0.87,2.08)	0.18
Tertile3	2.36(1.57,3.53)	< 0.0001
*P* for trend		< 0.0001
Model 3		
Continuous	1.26(1.09,1.45)	0.002
Categories		
Tertile1	ref	ref
Tertile2	1.27(0.81,1.98)	0.29
Tertile3	1.71(1.05,2.77)	0.03
*P* for trend		0.03

*HbA1c* Hemoglobin A1c, *HDL-C* High-density lipoprotein cholesterol, *BMI* Body mass index, *DM* Diabetes mellitus, *TC* Total cholesterol, *TG* Triglyceride, *FBG* Fasting blood glucose, *UA* Uric acid

Crude Model: no covariates were adjusted

Model 1: age, sex, education, residence, marital status, BMI were adjusted

Model 2: age, sex, education, residence, marital status, BMI, smoking, and drinking were adjusted

Model 3: age, sex, education, residence, marital status, BMI, smoking, drinking, hypertension, DM, dyslipidemia, chronic lung disease, heart disease, TC, TG, FBG, UA, and hemoglobin were adjusted

### Association between cumulative mean HbA1c/HDL-C and new stroke incidence

**Fig. 3:**
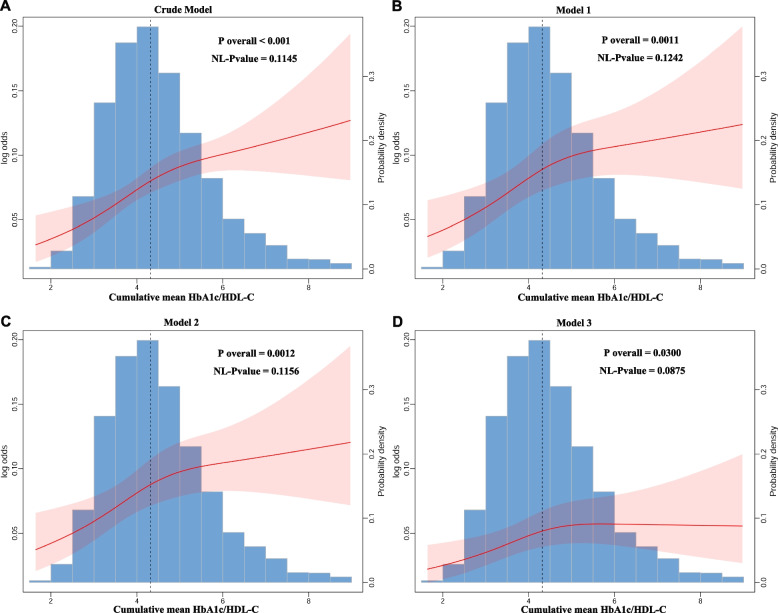
RCS curves are shown to reflect the dose–response association between cumulative mean HbA1c/HDL-C and new stroke incidence with different covariate adjustments. Odds ratios were represented by red solid lines, and 95% confidence intervals by red shaded areas. Abbreviations: RCS, Restricted cubic spline; HbA1c, hemoglobin A1c; HDL-C, high-density lipoprotein cholesterol

A consistent detrimental effect of cumulative mean HbA1c/HDL-C (2011–2015) on new stroke incidence in 2015–2018 is presented in Table 4. An increase in cumulative mean HbA1c/HDL-C was linked to a higher likelihood of new stroke incidence (OR:1.21, 95% CI: 1.12–1.33) in the crude model. After adjusting for various confounding factors, the connection weakened but remained significant, with ORs (95% CI) of 1.18 (1.08–1.29) for Model 1, 1.18 (1.07–1.30) for Model 2, and 1.14 (1.01–1.29) for Model 3. Next, cumulative mean HbA1c/HDL-C values were categorized into tertiles. The fully adjusted ORs were 1.65 (95% CI: 1.21–2.24) for T2 and 1.54 (95% CI: 1.08–2.19) for T3. The RCS analysis suggested a predominantly linear relationship between cumulative mean HbA1c/HDL-C levels and new stroke incidence (*p* for nonlinearity > 0.05) (Fig. 3). However, the segmented regression identified a potential saturation effect at cumulative mean HbA1c/HDL-C level of ≥ 4.42 (*p* for Log-likelihood ratio < 0.001). When the cumulative mean HbA1c/HDL-C level was < 4.42, the OR (95% CI) for stroke risk was 1.452 (1.005–2.097, *p* = 0.047). However, when the HbA1c/HDL-C level was ≥ 4.42, the OR (95% CI) was 1.036 (0.851–1.262, *p* = 0.723).

**Table 4:** The longitudinal study that exploring the associations between cumulative mean HbA1c/HDL-C (2011–2015) and new stroke incidence in 2015–2018

Cumulative mean HbA1c/HDL-C	New stroke incidence	
	OR (95% CI)	*P* value
Crude Model		
Continuous	1.21(1.12,1.33)	< 0.0001
Categories		
Tertile1	ref	ref
Tertile2	1.68(1.25,2.25)	< 0.001
Tertile3	1.95(1.46,2.60)	< 0.0001
*P* for trend		< 0.0001
Model 1		
Continuous	1.18(1.08,1.29)	< 0.001
Categories		
Tertile1	ref	ref
Tertile2	1.61(1.19,2.18)	0.002
Tertile3	1.78(1.32,2.41)	< 0.001
*P* for trend		< 0.001
Model 2		
Continuous	1.18(1.07,1.30)	< 0.001
Categories		
Tertile1	ref	ref
Tertile2	1.62(1.20,2.19)	0.002
Tertile3	1.79(1.32,2.42)	< 0.001
*P* for trend		< 0.001
Model 3		
Continuous	1.14(1.01,1.29)	0.04
Categories		
Tertile1	ref	ref
Tertile2	1.65(1.21,2.24)	0.002
Tertile3	1.54(1.08,2.19)	0.02
*P* for trend		0.02

*HbA1c* Hemoglobin A1c, *HDL-C* High-density lipoprotein cholesterol, *BMI* Body mass index, *DM* Diabetes mellitus, *TC* Total cholesterol, *TG* Triglyceride, *FBG* Fasting blood glucose, *UA* Uric acid

Crude Model: no covariates were adjusted

Model 1: age, sex, education, residence, marital status, BMI were adjusted

Model 2: age, sex, education, residence, marital status, BMI, smoking, and drinking were adjusted

Model 3: age, sex, education, residence, marital status, BMI, smoking, drinking, hypertension, DM, dyslipidemia, chronic lung disease, heart disease, TC, TG, FBG, UA, and hemoglobin were adjusted

### Predictive value of HbA1c/HDL-C in stroke risk

**Fig. 4:**
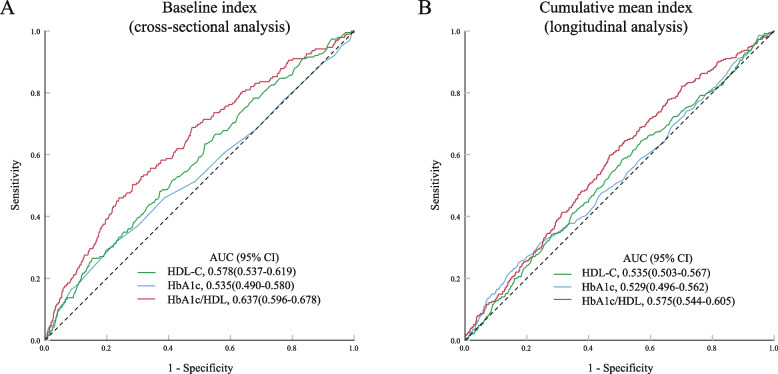
Predictive performance of the HbA1c, HDL-C, and HbA1c/HDL-C for stroke risk. HbA1c, hemoglobin A1c; HDL-C, high-density lipoprotein cholesterol; AUC, area under the curve; 95% CI, 95% confidence intervals

In the cross-sectional analysis (Fig. 4A), the ROC curve indicated an AUC of 0.637(0.596–0.678) for HbA1c/HDL-C, which was higher than that of individual HDL-C and HbA1c. The predictive value of HbA1c/HDL-C was significantly enhanced compared to HDL-C (NRI: 0.379, 95% CI: 0.235–0.523; IDI: 0.005, 95% CI: 0.003–0.007, *p* < 0.05) and HbA1c (NRI: 0.436, 95% CI: 0.294–0.579; IDI: 0.005, 95% CI: 0.003–0.007, *p* < 0.05). In the longitudinal analysis (Fig. 4B), the AUC for the cumulative mean HbA1c/HDL-C (0.575, 95% CI 0.544–0.605) showed a significant improvement compared to the traditional cumulative mean HDL-C and HbA1c indices. The ability to discriminate and risk reclassification were also significantly improved, with the NRI of 0.149 (95% CI 0.038–0.259, *p* < 0.05), IDI of 0.003 (95% CI 0.001–0.005, *p* < 0.05) compared with cumulative mean HDL-C; and the NRI of 0.190 (95% CI 0.080–0.301, *p* < 0.05), IDI of 0.003 (95% CI 0.002–0.005, *p* < 0.05) compared with cumulative mean HbA1c.

### Subgroup analysis

**Fig. 5:**
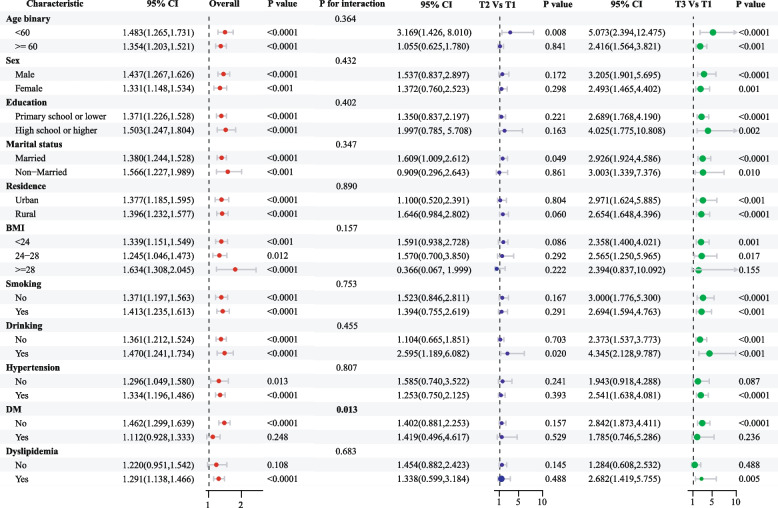
Subgroup and interaction analyses of the association between HbA1c/HDL-C (both continuous and categorical) and stroke risk. Abbreviations: CI, confidence interval; BMI, body mass index; DM, diabetes mellitus; HbA1c, hemoglobin A1c; HDL-C, high-density lipoprotein cholesterol

**Fig. 6:**
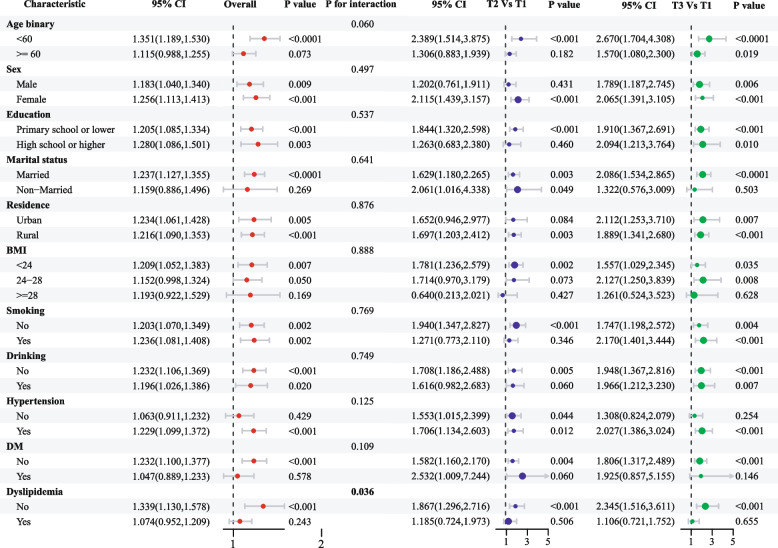
Subgroup and interaction analyses of the association between cumulative mean HbA1c/HDL-C (both continuous and categorical) and new stroke incidence. Abbreviations: CI, confidence interval; BMI, body mass index; DM, diabetes mellitus; HbA1c, hemoglobin A1c; HDL-C, high-density lipoprotein cholesterol

In the current investigation, subgroup analyses combined with interaction tests were performed to evaluate whether the relationship between HbA1c/HDL-C and stroke risk was influenced by potential risk factors. Stratification factors, including age, sex, education, marital status, residence, BMI, smoking, drinking, and hypertension, did not alter the correlation between continuous HbA1c/HDL-C and stroke risk in the cross-sectional study (Fig. 5 and Supplementary Table 6). As shown in the forest plot for HbA1c/HDL-C tertiles, similar findings were observed in the subgroup analysis for T3 vs. T1. It is noteworthy that, significant interactions were identified in the subgroup analyses for DM. In the DM group, the OR was 1.112 (95%CI: 0.928–1.333, *p* = 0.248), whereas in the non-DM group, the OR was 1.462 (95%CI: 1.299–1.639, *p* < 0.001). These results indicate that the association between HbA1c/HDL-C and stroke risk was more pronounced in individuals without DM. In the longitudinal study, the association between cumulative mean HbA1c/HDL-C and new stroke incidence remained consistent across subgroups based on sex, education, residence, smoking and drinking behavior (Fig. 6 and Supplementary Table 7). Similar findings from the subgroup analysis were observed for T3 vs. T1. The correlation between cumulative mean HbA1c/HDL-C and new stroke incidence was modified only by stratification for dyslipidemia.

## Discussion

We conducted a comprehensive cross-sectional and longitudinal analysis employing CHARLS datasets to determine the association between HbA1c/HDL-C and stroke. We identified that a higher HbA1c/HDL-C levels were associated with an increased risk of stroke at baseline. Smooth curve analyses confirmed a consistent linear trend, even after adjusting for confounding factors. Furthermore, our study demonstrated a positive relationship between cumulative mean HbA1c/HDL-C level and risk of new stroke incidence, with a potential saturation effect at a threshold of 4.42. The predictive value of the HbA1c/HDL-C in stroke risk assessment have been significantly improved compared to the traditional HDL-C and HbA1c. These findings were robust whether HbA1c/HDL-C was treated as a continuous or categorical variable. Subgroup analyses across most stratification factors further supported these findings.

Our findings demonstrated a significant positive correlation between both baseline and cumulative HbA1c/HDL-C levels and the risk of stroke. This is consistent with a meta-analysis by Mitsios et al., which included 29 studies with 532,799 participants. Their research found that higher HbA1c levels were associated with an elevated risk of stroke in individuals with DM, and a higher risk of ischemic stroke in those without DM [31]. The causal relationship between HDL-C and stroke risk remains an ongoing debate, particularly in Mendelian randomization studies. While HDL-C is generally protective against ischemic stroke, its role in hemorrhagic stroke is less clear, with some studies suggesting a neutral or even detrimental effect [32–34]. This indicates that the impact of HDL-C may differ across various stroke subtypes, which we did not distinguish in our current study. The failure to differentiate between stroke subtypes could influence the interpretation of HDL-C’s role in stroke risk. Future research incorporating detailed stroke subtype information is needed to better understand the specific impact of HDL-C on different stroke types and refine preventive strategies.

Segmented regression analysis notably identifies a potential saturation effect at a threshold of 4.42 in the relationship between cumulative mean HbA1c/HDL-C levels and new stroke incidence. This suggests that the relationship may deviate from strict linearity across the entire range of HbA1c/HDL-C values. The observed saturation effect likely reflects underlying biological mechanisms, such as advanced vascular damage or metabolic exhaustion, which may attenuate the incremental impact of further increases in HbA1c/HDL-C. Recent evidence further highlights the complex, non-linear relationships between glycolipid metabolism and stroke outcomes. Several studies have reported a U-shaped relationship between baseline HbA1c [35], HDL-C levels [36] and stroke risk, where stroke risk initially declines with increasing levels of HbA1c and HDL-C but rises again beyond certain thresholds [35, 36]. Similar patterns were observed when analyzing mean HbA1c and HDL-C levels over extended follow-up periods [35, 36]. Furthermore Chen et al. [37] identified a U-shaped association between LDL-C levels and post-stroke mortality, with the lowest risk observed at an LDL-C level of 2.67 mmol/L. These findings align with our observation of a potential saturation effect and underscore the importance of incorporating non-linear models when examining glycolipid metabolism and stroke risk. Identifying optimal HbA1c/HDL-C levels for stroke prevention could enhance risk stratification and inform more precise intervention strategies.

Our research presented the pioneer to explore the collective effect of HbA1c/HDL-C on stroke risk. A notable strength of this study is our use of laboratory data from 2011 and 2015 to calculate potential fluctuations in HbA1c/HDL-C levels during the follow-up period. The cumulative mean HbA1c/HDL-C represents long-term glycolipid metabolic status rather than a specific time point, thereby minimizing measurement errors and individual variability, thus providing more reliable clinical evidence [38, 39]. Prior studies have utilized other biological indices to reflect glycolipid homeostasis. Specifically, Wu et al. published a study on the association between the triglyceride glucose (TyG) index and stroke risk, a combined index derived from TG and glucose. Their research indicated that poorer control of the TyG index was associated with a higher likelihood of new stroke incidence [40]. In contrast to the short-term glucose indicator used in the TyG index, HbA1c—a long-term marker of blood glucose control—may provide more stable and clinically significant insights for stroke risk stratification. However, to date, no studies have directly compared the predictive performance of various glycolipid metabolism indicators for new-onset stroke. Our ROC analysis demonstrated that the HbA1c/HDL-C ratio significantly outperformed traditional HDL-C and HbA1c indices in predicting stroke risk. Future studies are warranted to further evaluate these indices and identify the most sensitive and accurate tool for risk estimation. Given its simplicity and accessibility, the HbA1c/HDL-C could be incorporated into community-based screening programs on a larger scale. By enabling early identification and personalized management of at-risk individuals, this marker could improve outcomes and reduce the stroke burden. However, further prospective studies and clinical trials are needed to validate its predictive accuracy and establish evidence-based thresholds for clinical decision-making.

In addition to glycolipid metabolism indicators, previous studies have extensively explored the relationships between other anthropometric measures, hematological markers, and stroke risk, providing valuable evidence. For instance, a longitudinal cohort conducted by Li et al. [41–43], which included 8,031 elderly individuals aged over 60 years, demonstrated that the weight-adjusted waist index, sarcopenia index, and systemic inflammation response index were significantly associated with the risks of both ischemic and hemorrhagic stroke in hypertensive patients. While both Li et al.’s study and ours adopted large-scale longitudinal cohort designs, several notable differences exist. Li et al.’s cohort focused on elderly hypertensive individuals from the Xinjiang region, with an older age demographic and a detailed investigation of different stroke subtypes. In contrast, the CHARLS cohort utilized in our study involved participants from 28 provinces across China. However, this cohort is limited by the lack of detailed information on stroke subtypes and the presence of unmeasured confounders. Future longitudinal studies should integrate these variables to gain a more comprehensive understanding of the relationship between glycolipid metabolism and stroke incidence.

Subgroup analysis revealed a consistent association between both baseline and cumulative HbA1c/HDL-C levels and stroke risk across most stratification factors, reinforcing the robustness and credibility of our findings and highlighting their relevance to a broader population. Notably, the association was more pronounced in individuals without DM than in those with DM. This disparity may be partly attributed to the influence of glucose- and lipid-lowering therapies, which could modify the determination of HbA1c/HDL-C levels [40]. These therapies are well-established to reduce stroke risk by improving metabolic control and vascular health [44], potentially attenuating the observed relationship between HbA1c/HDL-C and stroke risk in diabetic populations. Furthermore, patients with a longer disease duration or complications, such as diabetic nephropathy or retinopathy, are likely to experience greater vascular damage and metabolic dysregulation, which could alter the predictive value of HbA1c/HDL-C for stroke risk. Unfortunately, our study lacked detailed relevant data, limiting our ability to explore these effects. Our findings underscore the importance of HbA1c/HDL-C monitoring as part of primary stroke prevention strategies, particularly in non-diabetic individuals. Future studies should focus on personalized risk stratification in diabetic individuals, considering factors such as disease duration, treatment regimens, and the presence of complications. Additionally, our subgroup analyses were not adjusted for multiple comparisons, which increases the likelihood of Type I errors. This emphasizes the need for cautious interpretation and underscores the exploratory nature of these analyses.

The potential mechanisms underlying the association between HbA1c/HDL-C and stroke incidence are multifaceted, with several possible explanations. First, the composite index HbA1c/HDL-C reflects the balance between glucose and lipid metabolism, which is strongly linked to atherosclerosis, the primary etiology of stroke [22]. Previous studies have demonstrated that dysregulation of glycolipid metabolism can lead to vascular endothelial dysfunction, platelet hyperactivation, the release of vasoactive substances, foam cell aggregation, vulnerable plaque formation, and ultimately, stroke development [45–48]. Second, emerging evidence indicates that individuals with abnormal glycolipid metabolism exhibit elevated levels of inflammatory markers, such as high-sensitivity C-reactive protein, interleukin 6, and tumor necrosis factor α, as well as increased oxidative stress markers, including catalase and nitric oxide [49]. Disruption of glycolipid metabolism induces a state of low-grade inflammation, mitochondrial dysfunction, and oxidative stress, which collectively damage vascular endothelial and smooth muscle cells [50]. Third, lipotoxicity and glucotoxicity may directly impair neurons and cerebral microvessels, reduce cerebrovascular reserve, and disrupt hemodynamic regulation, thereby elevating the risk of stroke [51–53]. Although the exact mechanistic link between the HbA1c/HDL-C and stroke remains unclear, future combined interventions targeting both metabolic pathways may represent a potential therapeutic strategy for reducing stroke risk in patients with an elevated HbA1c/HDL-C level.

Our study has multiple strengths. To the best of our knowledge, this is the first study to comprehensively investigate the relationship between HbA1c/HDL-C (both continuous and categorical) and stroke risk, combining a baseline cross-sectional analysis with a seven-year longitudinal follow-up. Additionally, we utilized cumulative mean parameters rather than single time points in the longitudinal analysis, providing a more accurate representation of long-term glycolipid metabolism [54]. Nevertheless, certain limitations should be acknowledged. Firstly, the diagnosis of stroke was primarily based on physician assessment and self-reports via questionnaires, which may introduce recall bias. However, this method has been validated as a reliable surrogate and is widely accepted in cohort studies [55]. Secondly, due to limitations in the database, detailed information regarding stroke subtypes was unavailable, preventing us from evaluating potential differential effects of HbA1c/HDL-C on hemorrhagic, ischemic, or cardioembolic strokes. Thirdly, despite adjusting for various covariates across different logistic models and conducting subgroup analyses to guarantee the robustness and reliability of our findings, we cannot completely rule out the influence of unmeasured or poorly measured confounders (such as physical activity, dietary habits, and medications). Fourthly, the lack of multiple comparisons in the subgroup analyses increases the risk of false-positive findings, and the results should therefore be interpreted with caution. Finally, since the substantial amount of missing data was not random, the final sample may underrepresent middle-aged and older Chinese individuals and introduce selection bias, thereby limiting the generalizability of our findings. Further investigations, incorporating additional covariates and diverse populations, are warranted to verify and expand upon our findings.

## Conclusions

Based on the well-established CHARLS cohort, we identified a positive correlation between both baseline and cumulative HbA1c/HDL-C levels and the risk of stroke in middle-aged and older individuals. These findings have significant public health implications, offering valuable insights for improving long-term stroke risk assessment and guiding early prevention strategies. Further prospective studies, incorporating more detailed data on stroke subtypes and potential confounders, are warranted to validate and expand upon these findings.

## Supplementary Information

Supplementary Material 1: Supplementary Table 1 Comparing baseline characteristics of responders and non-responders in the cross-sectional (2011) analysis.

Supplementary Material 2: Supplementary Table 2 Comparing baseline characteristics of responders and non-responders in the longitudinal (2011-2018) analysis.

Supplementary Material 3: Supplementary Table 3. Diagnostic steps for collinearity between HbA1C/HDL-C and other covariates.

Supplementary Material 4: Supplementary Table 4 Baseline population characteristics of study participants based on stroke from the cross-sectional study in 2011.

Supplementary Material 5: Supplementary Table 5 Baseline population characteristics of study participants based on new stroke incidence from the 7 years longitudinal study.

Supplementary Material 6: Supplementary Table 6 Subgroup analyses of the association between HbA1c/HDL-C (both continuous and categorical) and stroke risk from the cross-sectional study in 2011.

Supplementary Material 7: Supplementary Table 7 Subgroup analyses of the association between cumulative mean HbA1c/HDL-C (both continuous and categorical) and new stroke incidence from the 7 years longitudinal study.

Supplementary Material 8: Supplementary Figure 1 Distribution of the HbA1c/HDL-C in 2011 and cumulative mean HbA1c/HDL-C in 2011-2015. HbA1c, hemoglobin A1c; HDL-C, high-density lipoprotein cholesterol.

## Data Availability

The datasets supporting the conclusions of this article are available on the CHARLS website: http://charls.pku.edu.cn/.

## Abbreviations

AIC: Akaike information criterion
AUC: Area under the curve
BIC: Bayesian information criterion
BMI: Body mass index
BUN: Blood urea nitrogen
CHARLS: China Health and Retirement Longitudinal Study
CI: Confidence intervals
DM: Diabetes mellitus
FBG: Fasting blood glucose
HbA1c: Hemoglobin A1c
HDL-C: High-density lipoprotein cholesterol
IDI: Integrated discrimination improvement
LDL-C: Low-density lipoprotein cholesterol
NRI: Net reclassification index
OR: Odds ratios
RCS: Restricted cubic spline
ROC: Receiver operating characteristic
SD: Standard deviation
TC: Total cholesterol
TG: Triglyceride
TyG: Triglyceride glucose
UA: Uric acid
