# Supplementary material for: Association between the hemoglobin A1c/High-density lipoprotein cholesterol ratio and stroke incidence: a prospective nationwide cohort study in China
Source: Lipids Health Dis. 2025 Jan 25;24:25. doi: 10.1186/s12944-025-02438-4 (PMC11762894; doi:10.1186/s12944-025-02438-4)
Supplement: Supplementary file 1 — Supplementary Material 1: Supplementary Table 1 Comparing baseline characteristics of responders and non-responders in the cross-sectional (2011) analysis. [file 12944_2025_2438_MOESM1_ESM.docx]

**Supplementary Table 1** Comparing baseline characteristics of responders and non-responders in the cross-sectional (2011) analysis.

| Characteristic | Total (n=17708) | Responders  (n=8502) | Non-responders  (n=9206) | Statistic | P value |
| --- | --- | --- | --- | --- | --- |
| Age, year | 58.99 ± 10.16 | 58.91 ± 8.74 | 59.07 ± 11.31 | 1.07 | 0.28 |
| Female, n (%) | 9221(52.12) | 4540(53.40) | 4681(50.94) | 10.64 | **<0.01** |
| Education, n (%) |  |  |  | 98.69 | **<0.0001** |
| Primary school or lower | 11755(66.59) | 5973(70.25) | 5782(63.18) |  |  |
| Middle school or higher | 5898(33.41) | 2529(29.75) | 3369(36.82) |  |  |
| Marital status, n (%) |  |  |  | 39.66 | **<0.0001** |
| Married | 15417(87.22) | 7556(88.87) | 7861(85.70) |  |  |
| Non-Married | 2258(12.78) | 946(11.13) | 1312(14.30) |  |  |
| Residence, n (%) |  |  |  | 254.52 | **<0.0001** |
| Rural area | 10537(59.51) | 5581(65.64) | 4956(53.85) |  |  |
| Urban | 7168(40.49) | 2921(34.36) | 4247(46.15) |  |  |
| BMI, kg/m^2^ | 23.47 ± 3.89 | 23.53 ± 3.87 | 23.37 ± 3.93 | -2.34 | **0.02** |
| Smoking, n (%) | 6931(39.48) | 3348(39.38) | 3583(39.57) | 0.06 | 0.81 |
| Drinking, n (%) | 5767(32.86) | 2797(32.90) | 2970(32.82) | 0.01 | 0.93 |
| Hypertension, n (%) | 6600(37.59) | 3450(40.58) | 3150(34.78) | 62.70 | **<0.0001** |
| DM, n (%) | 2087(11.88) | 1152(13.55) | 935(10.31) | 43.59 | **<0.0001** |
| Dyslipidemia, n (%) | 5280(30.25) | 3380(39.76) | 1900(21.22) | 709.54 | **<0.0001** |
| Heart disease, n (%) | 2051(11.74) | 971(11.42) | 1080(12.05) | 1.60 | 0.21 |
| Chronic lung disease, n (%) | 1814(10.37) | 900(10.59) | 914(10.16) | 0.79 | 0.37 |
| Stroke, n (%) | 435(2.48) | 189(2.22) | 246(2.73) | 4.37 | **0.04** |
| Hemoglobin, g/dL | 14.38 ± 2.22 | 14.39 ± 2.21 | 14.38 ± 2.26 | -0.13 | 0.90 |
| TC, mg/dL | 192.97 ± 38.89 | 193.75 ± 38.19 | 190.85 ± 40.67 | -3.48 | **<0.001** |
| TG, mg/dL | 134.91 ± 110.26 | 128.15 ± 85.22 | 153.12 ± 157.81 | 8.44 | **<0.0001** |
| LDL-C, mg/dL | 115.99 ± 34.91 | 117.25 ± 34.58 | 112.60 ± 35.56 | -6.30 | **<0.0001** |
| FBG, mg/dL | 110.30 ± 37.33 | 108.55 ± 31.30 | 115.04 ± 49.86 | 6.81 | **<0.0001** |
| BUN, mg/dL | 15.75 ± 4.65 | 15.70 ± 4.48 | 15.89 ± 5.07 | 1.85 | 0.06 |
| Creatinine, mg/dL | 0.78 ± 0.24 | 0.78 ± 0.20 | 0.79 ± 0.32 | 2.43 | **0.02** |
| UA, mg/dL | 4.46 ± 1.27 | 4.44 ± 1.25 | 4.52 ± 1.32 | 3.04 | **<0.01** |
| HbA1c/HDL | 4.42 ± 1.86 | 4.25 ± 1.33 | 4.88 ± 2.82 | 11.96 | **<0.0001** |

**Notes:** BMI, body mass index; DM, diabetes mellitus; TC, total cholesterol; TG, triglyceride; LDL-C, low density lipoprotein cholesterol; FBG, fasting blood glucose; BUN, blood urea nitrogen; UA, uric acid; HbA1c, glycosylated hemoglobin A1c; HDL-C, high-density lipoprotein cholesterol.
