# Supplementary material for: Association between the hemoglobin A1c/High-density lipoprotein cholesterol ratio and stroke incidence: a prospective nationwide cohort study in China
Source: Lipids Health Dis. 2025 Jan 25;24:25. doi: 10.1186/s12944-025-02438-4 (PMC11762894; doi:10.1186/s12944-025-02438-4)
Supplement: Supplementary file 2 — Supplementary Material 2: Supplementary Table 2 Comparing baseline characteristics of responders and non-responders in the longitudinal (2011-2018) analysis. [file 12944_2025_2438_MOESM2_ESM.docx]

**Supplementary Table 2** Comparing baseline characteristics of responders and non-responders in the longitudinal (2011-2018) analysis.

| Characteristic | Total (n=6746) | Responders  (n=5165) | Non-responders  (n=1581) | Statistic | P value |
| --- | --- | --- | --- | --- | --- |
| Age, year | 58.32 ± 8.81 | 58.35 ± 8.24 | 58.21 ± 10.48 | -0.47 | 0.64 |
| Female, n (%) | 3787(56.18) | 2867(55.51) | 920(58.38) | 3.92 | 0.05 |
| Education, n (%) |  |  |  | 0.26 | 0.61 |
| Primary school or lower | 4733(70.19) | 3634(70.36) | 1099(69.65) |  |  |
| Middle school or higher | 2010(29.81) | 1531(29.64) | 479(30.35) |  |  |
| Marital status, n (%) |  |  |  | 8.39 | **<0.01** |
| Married | 6055(89.76) | 4667(90.36) | 1388(87.79) |  |  |
| Non-Married | 691(10.24) | 498(9.64) | 193(12.21) |  |  |
| Residence, n (%) |  |  |  | 14.12 | **<0.001** |
| Rural area | 4502(66.74) | 3509(67.94) | 993(62.81) |  |  |
| Urban | 2244(33.26) | 1656(32.06) | 588(37.19) |  |  |
| BMI, kg/m^2^ | 23.72 ± 3.86 | 23.68 ± 3.86 | 23.96 ± 3.81 | 2.05 | **0.04** |
| Smoking, n (%) | 2516(37.30) | 1936(37.48) | 580(36.69) | 0.30 | 0.59 |
| Drinking, n (%) | 2164(32.08) | 1692(32.76) | 472(29.85) | 4.55 | **0.03** |
| Hypertension, n (%) | 2619(38.83) | 1999(38.70) | 620(39.27) | 0.14 | 0.71 |
| DM, n (%) | 950(14.08) | 656(12.70) | 294(18.60) | 34.28 | **<0.0001** |
| Dyslipidemia, n (%) | 2753(40.89) | 2077(40.21) | 676(43.14) | 4.14 | **0.04** |
| Heart disease, n (%) | 781(11.65) | 553(10.71) | 228(14.82) | 19.12 | **<0.0001** |
| Chronic lung disease, n (%) | 685(10.19) | 513(9.93) | 172(11.04) | 1.49 | 0.22 |
| Hemoglobin, g/dL | 14.39 ± 2.20 | 14.39 ± 2.17 | 14.40 ± 2.29 | 0.29 | 0.77 |
| TC, mg/dL | 193.13 ± 38.36 | 193.52 ± 38.01 | 191.75 ± 39.53 | -1.53 | 0.13 |
| TG, mg/dL | 135.24 ± 110.72 | 130.58 ± 93.65 | 151.63 ± 155.51 | 4.94 | **<0.0001** |
| LDL-C, mg/dL | 116.09 ± 34.57 | 116.71 ± 34.37 | 113.87 ± 35.18 | -2.73 | **<0.01** |
| FBG, mg/dL | 109.92 ± 35.56 | 108.19 ± 30.92 | 116.04 ± 48.03 | 5.91 | **<0.0001** |
| BUN, mg/dL | 15.61 ± 4.37 | 15.64 ± 4.32 | 15.49 ± 4.52 | -1.11 | 0.27 |
| Creatinine, mg/dL | 0.76 ± 0.18 | 0.77 ± 0.18 | 0.76 ± 0.19 | -0.24 | 0.81 |
| UA, mg/dL | 4.37 ± 1.22 | 4.36 ± 1.21 | 4.39 ± 1.26 | 0.94 | 0.35 |
| HbA1c/HDL | 4.59 ± 1.46 | 4.48 ± 1.18 | 5.01 ± 2.14 | 8.87 | **<0.0001** |

**Notes:** BMI, body mass index; DM, diabetes mellitus; TC, total cholesterol; TG, triglyceride; LDL-C, low density lipoprotein cholesterol; FBG, fasting blood glucose; BUN, blood urea nitrogen; UA, uric acid; HbA1c, glycosylated hemoglobin A1c; HDL-C, high-density lipoprotein cholesterol.
