# Supplementary material for: Association between the hemoglobin A1c/High-density lipoprotein cholesterol ratio and stroke incidence: a prospective nationwide cohort study in China
Source: Lipids Health Dis. 2025 Jan 25;24:25. doi: 10.1186/s12944-025-02438-4 (PMC11762894; doi:10.1186/s12944-025-02438-4)
Supplement: Supplementary file 3 — Supplementary Material 3: Supplementary Table 3. Diagnostic steps for collinearity between HbA1C/HDL-C and other covariates. [file 12944_2025_2438_MOESM3_ESM.docx]

**Supplementary Table 3.** Diagnostic steps for collinearity between HbA1C/HDL-C and other covariates.

|  | **VIF** | |
| --- | --- | --- |
|  | Cross-sectional analysis | Longitudinal analysis |
| Age | 1.204 | 1.179 |
| Sex | 2.570 | 2.634 |
| Education | 1.182 | 1.167 |
| Marital status | 1.066 | 1.059 |
| Residence | 1.081 | 1.058 |
| BMI | 1.239 | 1.248 |
| Smoking | 1.978 | 1.985 |
| Drinking | 1.343 | 1.376 |
| Hypertension | 1.138 | 1.140 |
| DM | 1.582 | 1.626 |
| Dyslipidemia | 1.719 | 1.580 |
| Heart disease | 1.033 | 1.031 |
| Chronic lung disease | 1.060 | 1.055 |
| Hemoglobin | 1.195 | 1.187 |
| FBG | 1.649 | 1.678 |
| UA | 1.297 | 1.301 |
| TC | 1.483 | 1.394 |
| TG | 1.693 | 1.569 |
| HbA1c/HDL-C | 2.164 | 1.885 |

VIF = 1/(1-R^2^). The variables with VIF>5 will be regarded as collinear variables and cannot be included in the multiple regression model.

**Notes:** HbA1c, hemoglobin A1c; HDL-C, high-density lipoprotein cholesterol; VIF: variance inflation factor; BMI, body mass index; DM, diabetes mellitus; FBG, fasting blood glucose; UA, uric acid; TC, total cholesterol; TG, triglyceride.
