# Supplementary material for: Association between the hemoglobin A1c/High-density lipoprotein cholesterol ratio and stroke incidence: a prospective nationwide cohort study in China
Source: Lipids Health Dis. 2025 Jan 25;24:25. doi: 10.1186/s12944-025-02438-4 (PMC11762894; doi:10.1186/s12944-025-02438-4)
Supplement: Supplementary file 4 — Supplementary Material 4: Supplementary Table 4 Baseline population characteristics of study participants based on stroke from the cross-sectional study in 2011. [file 12944_2025_2438_MOESM4_ESM.docx]

**Supplementary Table 4** Baseline population characteristics of study participants based on stroke from the cross-sectional study in 2011.

| Characteristic | Total (n=8502) | Stroke | | Statistic | P value |
| --- | --- | --- | --- | --- | --- |
|  |  | No  (n=8313) | Yes  (n=189) |  |  |
| Age, year | 58.91 ± 8.74 | 58.82 ± 8.73 | 62.56 ± 8.23 | -6.15 | **<0.0001** |
| Age, n (%) |  |  |  | 32.04 | **<0.0001** |
| <60 | 4712(55.42) | 4646(55.89) | 66(34.92) |  |  |
| >=60 | 3790(44.58) | 3667(44.11) | 123(65.08) |  |  |
| Female, n (%) | 4540(53.40) | 4453(53.57) | 87(46.03) | 3.92 | 0.05 |
| Education, n (%) |  |  |  | 1.97 | 0.16 |
| Primary school or lower | 5973(70.25) | 5831(70.14) | 142(75.13) |  |  |
| Middle school or higher | 2529(29.75) | 2482(29.86) | 47(24.87) |  |  |
| Marital status, n (%) |  |  |  | 7.20 | **<0.01** |
| Married | 7556(88.87) | 7400(89.02) | 156(82.54) |  |  |
| Non-Married | 946(11.13) | 913(10.98) | 33(17.46) |  |  |
| Residence, n (%) |  |  |  | 3.21 | 0.07 |
| Rural area | 5581(65.64) | 5469(65.79) | 112(59.26) |  |  |
| Urban | 2921(34.36) | 2844(34.21) | 77(40.74) |  |  |
| BMI, kg/m^2^ |  |  |  | 9.46 | **<0.01** |
| <24 | 5822(68.48) | 5712(68.71) | 110(58.20) |  |  |
| 24-28 | 2267(26.66) | 2200(26.46) | 67(35.45) |  |  |
| >=28 | 413(4.86) | 401(4.82) | 12(6.35) |  |  |
| Smoking, n (%) | 3348(39.38) | 3255(39.16) | 93(49.21) | 7.40 | **<0.01** |
| Drinking, n (%) | 2797(32.90) | 2741(32.97) | 56(29.63) | 0.79 | 0.37 |
| Hypertension, n (%) | 3450(40.58) | 3305(39.76) | 145(76.72) | 103.18 | **<0.0001** |
| DM, n (%) | 1152(13.55) | 1106(13.30) | 46(24.34) | 18.28 | **<0.0001** |
| Dyslipidemia, n (%) | 3380(39.76) | 3265(39.28) | 115(60.85) | 35.01 | **<0.0001** |
| Heart disease, n (%) | 971(11.42) | 927(11.15) | 44(23.28) | 25.69 | **<0.0001** |
| Chronic lung disease, n (%) | 900(10.59) | 869(10.45) | 31(16.40) | 6.29 | **0.01** |
| Hemoglobin, g/dL | 14.39 ± 2.21 | 14.37 ± 2.20 | 14.92 ± 2.62 | -2.86 | **<0.01** |
| TC, mg/dL | 193.75 ± 38.19 | 193.69 ± 38.06 | 196.48 ± 43.66 | -0.87 | 0.39 |
| TG, mg/dL | 128.15 ± 85.22 | 127.69 ± 85.04 | 148.32 ± 90.94 | -3.09 | **<0.01** |
| LDL-C, mg/dL | 128.15 ± 85.22 | 127.69 ± 85.04 | 148.32 ± 90.94 | -3.09 | **<0.01** |
| FBG, mg/dL | 108.55 ± 31.30 | 108.42 ± 31.24 | 114.30 ± 33.38 | -2.40 | **0.02** |
| BUN, mg/dL | 15.70 ± 4.48 | 15.69 ± 4.47 | 15.90 ± 5.00 | -0.57 | 0.57 |
| Creatinine, mg/dL | 0.78 ± 0.20 | 0.78 ± 0.20 | 0.85 ± 0.23 | -4.27 | **<0.0001** |
| UA, mg/dL | 4.44 ± 1.25 | 4.43 ± 1.24 | 4.84 ± 1.43 | -3.90 | **<0.001** |
| HbA1c/HDL | 4.25 ± 1.33 | 4.23 ± 1.32 | 4.92 ± 1.51 | -6.23 | **<0.0001** |

**Notes:** HbA1c, glycosylated hemoglobin A1c; HDL-C, high-density lipoprotein cholesterol; BMI, body mass index; DM, diabetes mellitus; TC, total cholesterol; TG, triglyceride; LDL-C, low-density lipoprotein cholesterol; FBG, fasting blood glucose; BUN, blood urea nitrogen; UA, uric acid.
