# Supplementary material for: Association between the hemoglobin A1c/High-density lipoprotein cholesterol ratio and stroke incidence: a prospective nationwide cohort study in China
Source: Lipids Health Dis. 2025 Jan 25;24:25. doi: 10.1186/s12944-025-02438-4 (PMC11762894; doi:10.1186/s12944-025-02438-4)
Supplement: Supplementary file 5 — Supplementary Material 5: Supplementary Table 5 Baseline population characteristics of study participants based on new stroke incidence from the 7 years longitudinal study. [file 12944_2025_2438_MOESM5_ESM.docx]

**Supplementary Table 5** Baseline population characteristics of study participants based on new stroke incidence from the 7 years longitudinal study.

| Characteristic | Total (n=5165) | Stroke | | Statistic | P value |
| --- | --- | --- | --- | --- | --- |
|  |  | No  (n=4829) | Yes  (n=336) |  |  |
| Age, year | 58.35 ± 8.24 | 58.18 ± 8.23 | 60.74 ± 7.97 | -5.67 | **<0.0001** |
| Age, n (%) |  |  |  | 17.67 | **<0.0001** |
| <60 | 2942(56.96) | 2788(57.73) | 154(45.83) |  |  |
| >=60 | 2223(43.04) | 2041(42.27) | 182(54.17) |  |  |
| Female, n (%) | 2867(55.51) | 2680(55.50) | 187(55.65) | 0.00 | 1.00 |
| Education, n (%) |  |  |  | 2.23 | 0.14 |
| Primary school or lower | 3634(70.36) | 3385(70.10) | 249(74.11) |  |  |
| Middle school or higher | 1531(29.64) | 1444(29.90) | 87(25.89) |  |  |
| Marital status, n (%) |  |  |  | 7.27 | **<0.01** |
| Married | 4667(90.36) | 4378(90.66) | 289(86.01) |  |  |
| Non-Married | 498(9.64) | 451(9.34) | 47(13.99) |  |  |
| Residence, n (%) |  |  |  | 0.00 | 0.98 |
| Rural area | 3509(67.94) | 3280(67.92) | 229(68.15) |  |  |
| Urban | 1656(32.06) | 1549(32.08) | 107(31.85) |  |  |
| BMI, kg/m^2^ |  |  |  | 12.61 | **<0.01** |
| <24 | 3004(58.16) | 2837(58.75) | 167(49.70) |  |  |
| 24-28 | 1745(33.79) | 1615(33.44) | 130(38.69) |  |  |
| >=28 | 416(8.05) | 377(7.81) | 39(11.61) |  |  |
| Smoking, n (%) | 1936(37.48) | 1801(37.30) | 135(40.18) | 0.99 | 0.32 |
| Drinking, n (%) | 1692(32.76) | 1584(32.80) | 108(32.14) | 0.04 | 0.85 |
| Hypertension, n (%) | 1999(38.70) | 1791(37.09) | 208(61.90) | 80.51 | **<0.0001** |
| DM, n (%) | 656(12.70) | 590(12.22) | 66(19.64) | 14.96 | **<0.001** |
| Dyslipidemia, n (%) | 2077(40.21) | 1906(39.47) | 171(50.89) | 16.58 | **<0.0001** |
| Heart disease, n (%) | 553(10.71) | 486(10.06) | 67(19.94) | 31.03 | **<0.0001** |
| Chronic lung disease, n (%) | 513(9.93) | 475(9.84) | 38(11.31) | 0.61 | 0.44 |
| Hemoglobin, g/dL | 14.39 ± 2.17 | 14.38 ± 2.17 | 14.50 ± 2.23 | -0.98 | 0.33 |
| TC, mg/dL | 193.52 ± 38.01 | 193.28 ± 38.10 | 197.05 ± 36.64 | -1.82 | 0.07 |
| TG, mg/dL | 130.58 ± 93.65 | 129.76 ± 93.62 | 142.25 ± 93.39 | -2.37 | **0.02** |
| LDL-C, mg/dL | 116.71 ± 34.37 | 116.46 ± 34.27 | 120.35 ± 35.62 | -1.94 | 0.05 |
| FBG, mg/dL | 108.19 ± 30.92 | 107.95 ± 30.93 | 111.63 ± 30.61 | -2.13 | **0.03** |
| BUN, mg/dL | 15.64 ± 4.32 | 15.63 ± 4.31 | 15.75 ± 4.51 | -0.48 | 0.63 |
| Creatinine, mg/dL | 0.77 ± 0.18 | 0.76 ± 0.18 | 0.78 ± 0.17 | -1.45 | 0.15 |
| UA, mg/dL | 4.36 ± 1.21 | 4.35 ± 1.21 | 4.44 ± 1.21 | -1.21 | 0.23 |
| Cumulative mean HbA1c/HDL-C | 4.48 ± 1.18 | 4.46 ± 1.17 | 4.76 ± 1.25 | -4.27 | **<0.0001** |

**Notes:** HbA1c, glycosylated hemoglobin A1c; HDL-C, high-density lipoprotein cholesterol; BMI, body mass index; DM, diabetes mellitus; TC, total cholesterol; TG, triglyceride; LDL-C, low density lipoprotein cholesterol; FBG, fasting blood glucose; BUN, blood urea nitrogen; UA, uric acid.
