# Supplementary material for: Association between the hemoglobin A1c/High-density lipoprotein cholesterol ratio and stroke incidence: a prospective nationwide cohort study in China
Source: Lipids Health Dis. 2025 Jan 25;24:25. doi: 10.1186/s12944-025-02438-4 (PMC11762894; doi:10.1186/s12944-025-02438-4)
Supplement: Supplementary file 6 — Supplementary Material 6: Supplementary Table 6 Subgroup analyses of the association between HbA1c/HDL-C (both continuous and categorical) and stroke risk from the cross-sectional study in 2011. [file 12944_2025_2438_MOESM6_ESM.docx]

**Supplementary Table 6** Subgroup analyses of the association between HbA1c/HDL-C (both continuous and categorical) and stroke risk from the cross-sectional study in 2011.

| Subgroup | OR (95% CI) | P value | P for interaction | T1 | T2 vs. T1 | P value | T3 vs. T1 | P value | P for trend |
| --- | --- | --- | --- | --- | --- | --- | --- | --- | --- |
| **Age** |  |  | 0.364 |  |  |  |  |  |  |
| <60 | 1.483(1.265,1.731) | <0.0001 |  | ref | 3.169(1.426, 8.010) | 0.008 | 5.073(2.394,12.475) | <0.0001 | <0.0001 |
| >= 60 | 1.354(1.203,1.521) | <0.0001 |  | ref | 1.055(0.625,1.780) | 0.841 | 2.416(1.564,3.821) | <0.001 | <0.0001 |
| **Sex** |  |  | 0.432 |  |  |  |  |  |  |
| Male | 1.437(1.267,1.626) | <0.0001 |  | ref | 1.537(0.837,2.897) | 0.172 | 3.205(1.901,5.695) | <0.0001 | <0.0001 |
| Female | 1.331(1.148,1.534) | <0.001 |  | ref | 1.372(0.760,2.523) | 0.298 | 2.493(1.465,4.402) | 0.001 | <0.001 |
| **Education** |  |  | 0.402 |  |  |  |  |  |  |
| Primary school or lower | 1.371(1.226,1.528) | <0.0001 |  | ref | 1.350(0.837,2.197) | 0.221 | 2.689(1.768,4.190) | <0.0001 | <0.0001 |
| High school or higher | 1.503(1.247,1.804) | <0.0001 |  | ref | 1.997(0.785, 5.708) | 0.163 | 4.025(1.775,10.808) | 0.002 | <0.001 |
| **Marital status** |  |  | 0.347 |  |  |  |  |  |  |
| Married | 1.380(1.244,1.528) | <0.0001 |  | ref | 1.609(1.009,2.612) | 0.049 | 2.926(1.924,4.586) | <0.0001 | <0.0001 |
| Non-Married | 1.566(1.227,1.989) | <0.001 |  | ref | 0.909(0.296,2.643) | 0.861 | 3.003(1.339,7.376) | 0.010 | 0.006 |
| **Residence** |  |  | 0.890 |  |  |  |  |  |  |
| Urban | 1.377(1.185,1.595) | <0.0001 |  | ref | 1.100(0.520,2.391) | 0.804 | 2.971(1.624,5.885) | <0.001 | <0.0001 |
| Rural | 1.396(1.232,1.577) | <0.0001 |  | ref | 1.646(0.984,2.802) | 0.060 | 2.654(1.648,4.396) | <0.0001 | <0.0001 |
| **BMI** |  |  | 0.157 |  |  |  |  |  |  |
| <24 | 1.591(0.938,2.728) | <0.0001 |  | ref | 1.591(0.938,2.728) | 0.086 | 2.358(1.400,4.021) | 0.001 | 0.001 |
| 24-28 | 1.570(0.700,3.850) | <0.001 |  | ref | 1.570(0.700,3.850) | 0.292 | 2.565(1.250,5.965) | 0.017 | 0.008 |
| >=28 | 0.366(0.067,1.999) | 0.002 |  | ref | 0.366(0.067,1.999) | 0.222 | 2.394(0.837,10.092) | 0.155 | 0.003 |
| **Smoking** |  |  | 0.753 |  |  |  |  |  |  |
| No | 1.371(1.197,1.563) | <0.0001 |  | ref | 1.523(0.846,2.811) | 0.167 | 3.000(1.776,5.300) | <0.0001 | <0.0001 |
| Yes | 1.413(1.235,1.613) | <0.0001 |  | ref | 1.394(0.755,2.619) | 0.291 | 2.694(1.594,4.763) | <0.001 | <0.001 |
| **Drinking** |  |  | 0.455 |  |  |  |  |  |  |
| No | 1.361(1.212,1.524) | <0.0001 |  | ref | 1.104(0.665,1.851) | 0.703 | 2.373(1.537,3.773) | <0.001 | <0.0001 |
| Yes | 1.470(1.241,1.734) | <0.0001 |  | ref | 2.595(1.189,6.082) | 0.020 | 4.345(2.128,9.787) | <0.001 | <0.0001 |
| **Hypertension** |  |  | 0.807 |  |  |  |  |  |  |
| No | 1.296(1.049,1.580) | 0.013 |  | ref | 1.585(0.740,3.522) | 0.241 | 1.943(0.918,4.288) | 0.087 | 0.087 |
| Yes | 1.334(1.196,1.486) | <0.0001 |  | ref | 1.253(0.750,2.125) | 0.393 | 2.541(1.638,4.081) | <0.0001 | <0.0001 |
| **DM** |  |  | **0.013** |  |  |  |  |  |  |
| No | 1.462(1.299,1.639) | <0.0001 |  | ref | 1.402(0.881,2.253) | 0.157 | 2.842(1.873,4.411) | <0.0001 | <0.0001 |
| Yes | 1.112(0.928,1.333) | 0.248 |  | ref | 1.419(0.496,4.617) | 0.529 | 1.785(0.746,5.286) | 0.236 | 0.209 |
| **Dyslipidemia** |  |  | 0.683 |  |  |  |  |  |  |
| No | 1.220(0.951,1.542) | 0.108 |  | ref | 1.454(0.882,2.423) | 0.145 | 1.284(0.608,2.532) | 0.488 | 0.280 |
| Yes | 1.291(1.138,1.466) | <0.0001 |  | ref | 1.338(0.599,3.184) | 0.488 | 2.682(1.419,5.755) | 0.005 | <0.001 |

**Notes:** HbA1c, glycosylated hemoglobin A1c; HDL-C, high-density lipoprotein cholesterol; BMI, body mass index; DM, diabetes mellitus.
