# Supplementary material for: Association between the hemoglobin A1c/High-density lipoprotein cholesterol ratio and stroke incidence: a prospective nationwide cohort study in China
Source: Lipids Health Dis. 2025 Jan 25;24:25. doi: 10.1186/s12944-025-02438-4 (PMC11762894; doi:10.1186/s12944-025-02438-4)
Supplement: Supplementary file 7 — Supplementary Material 7: Supplementary Table 7 Subgroup analyses of the association between cumulative mean HbA1c/HDL-C (both continuous and categorical) and new stroke incidence from the 7 years longitudinal study. [file 12944_2025_2438_MOESM7_ESM.docx]

**Supplementary Table 7** Subgroup analyses of the association between cumulative mean HbA1c/HDL-C (both continuous and categorical) and new stroke incidence from the 7 years longitudinal study.

| Subgroup | OR (95% CI) | P value | P for interaction | T1 | T2 vs. T1 | P value | T3 vs. T1 | P value | P for trend |
| --- | --- | --- | --- | --- | --- | --- | --- | --- | --- |
| **Age** |  |  | 0.060 |  |  |  |  |  |  |
| <60 | 1.351(1.189,1.530) | <0.0001 |  | ref | 2.389(1.514,3.875) | <0.001 | 2.670(1.704,4.308) | <0.0001 | <0.0001 |
| >= 60 | 1.115(0.988,1.255) | 0.073 |  | ref | 1.306(0.883,1.939) | 0.182 | 1.570(1.080,2.300) | 0.019 | 0.019 |
| **Sex** |  |  | 0.497 |  |  |  |  |  |  |
| Male | 1.183(1.040,1.340) | 0.009 |  | ref | 1.202(0.761,1.911) | 0.431 | 1.789(1.187,2.745) | 0.006 | 0.004 |
| Female | 1.256(1.113,1.413) | <0.001 |  | ref | 2.115(1.439,3.157) | <0.001 | 2.065(1.391,3.105) | <0.001 | <0.001 |
| **Education** |  |  | 0.537 |  |  |  |  |  |  |
| Primary school or lower | 1.205(1.085,1.334) | <0.001 |  | ref | 1.844(1.320,2.598) | <0.001 | 1.910(1.367,2.691) | <0.001 | <0.001 |
| High school or higher | 1.280(1.086,1.501) | 0.003 |  | ref | 1.263(0.683,2.380) | 0.460 | 2.094(1.213,3.764) | 0.010 | 0.006 |
| **Marital status** |  |  | 0.641 |  |  |  |  |  |  |
| Married | 1.237(1.127,1.355) | <0.0001 |  | ref | 1.629(1.180,2.265) | 0.003 | 2.086(1.534,2.865) | <0.0001 | <0.0001 |
| Non-Married | 1.159(0.886,1.496) | 0.269 |  | ref | 2.061(1.016,4.338) | 0.049 | 1.322(0.576,3.009) | 0.503 | 0.426 |
| **Residence** |  |  | 0.876 |  |  |  |  |  |  |
| Urban | 1.234(1.061,1.428) | 0.005 |  | ref | 1.652(0.946,2.977) | 0.084 | 2.112(1.253,3.710) | 0.007 | 0.006 |
| Rural | 1.216(1.090,1.353) | <0.001 |  | ref | 1.697(1.203,2.412) | 0.003 | 1.889(1.341,2.680) | <0.001 | <0.001 |
| **BMI** |  |  | 0.888 |  |  |  |  |  |  |
| <24 | 1.209(1.052,1.383) | 0.007 |  | ref | 1.781(1.236,2.579) | 0.002 | 1.557(1.029,2.345) | 0.035 | 0.017 |
| 24-28 | 1.152(0.998,1.324) | 0.050 |  | ref | 1.714(0.970,3.179) | 0.073 | 2.127(1.250,3.839) | 0.008 | 0.008 |
| >=28 | 1.193(0.922,1.529) | 0.169 |  | ref | 0.640(0.213,2.021) | 0.427 | 1.261(0.524,3.523) | 0.628 | 0.311 |
| **Smoking** |  |  | 0.769 |  |  |  |  |  |  |
| No | 1.203(1.070,1.349) | 0.002 |  | ref | 1.940(1.347,2.827) | <0.001 | 1.747(1.198,2.572) | 0.004 | 0.005 |
| Yes | 1.236(1.081,1.408) | 0.002 |  | ref | 1.271(0.773,2.110) | 0.346 | 2.170(1.401,3.444) | <0.001 | <0.001 |
| **Drinking** |  |  | 0.749 |  |  |  |  |  |  |
| No | 1.232(1.106,1.369) | <0.001 |  | ref | 1.708(1.186,2.488) | 0.005 | 1.948(1.367,2.816) | <0.001 | <0.001 |
| Yes | 1.196(1.026,1.386) | 0.020 |  | ref | 1.616(0.982,2.683) | 0.060 | 1.966(1.212,3.230) | 0.007 | 0.007 |
| **Hypertension** |  |  | 0.125 |  |  |  |  |  |  |
| No | 1.063(0.911,1.232) | 0.429 |  | ref | 1.553(1.015,2.399) | 0.044 | 1.308(0.824,2.079) | 0.254 | 0.232 |
| Yes | 1.229(1.099,1.372) | <0.001 |  | ref | 1.706(1.134,2.603) | 0.012 | 2.027(1.386,3.024) | <0.001 | <0.001 |
| **DM** |  |  | 0.109 |  |  |  |  |  |  |
| No | 1.232(1.100,1.377) | <0.001 |  | ref | 1.582(1.160,2.170) | 0.004 | 1.806(1.317,2.489) | <0.001 | <0.001 |
| Yes | 1.047(0.889,1.233) | 0.578 |  | ref | 2.532(1.009,7.244) | 0.060 | 1.925(0.857,5.155) | 0.146 | 0.338 |
| **Dyslipidemia** |  |  | **0.036** |  |  |  |  |  |  |
| No | 1.339(1.130,1.578) | <0.001 |  | ref | 1.867(1.296,2.716) | <0.001 | 2.345(1.516,3.611) | <0.001 | <0.0001 |
| Yes | 1.074(0.952,1.209) | 0.243 |  | ref | 1.185(0.724,1.973) | 0.506 | 1.106(0.721,1.752) | 0.655 | 0.786 |

**Notes:** HbA1c, glycosylated hemoglobin A1c; HDL-C, high-density lipoprotein cholesterol; BMI, body mass index; DM, diabetes mellitus.
