# Supplementary figures and images for: Association between the hemoglobin A1c/High-density lipoprotein cholesterol ratio and stroke incidence: a prospective nationwide cohort study in China
Source: Lipids Health Dis. 2025 Jan 25;24:25. doi: 10.1186/s12944-025-02438-4 (PMC11762894; doi:10.1186/s12944-025-02438-4)

A

Histogram of Normal Distribution

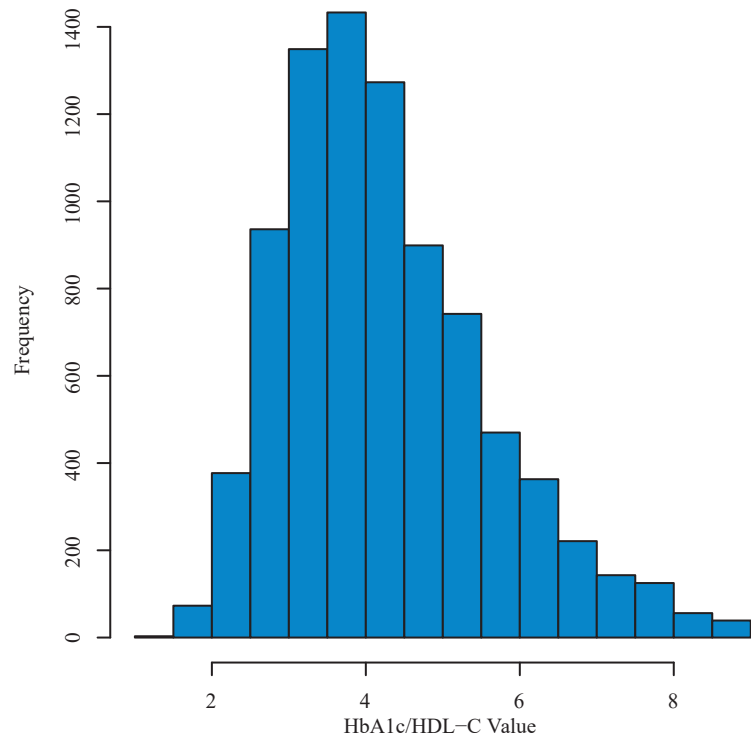

B

Histogram of Normal Distribution

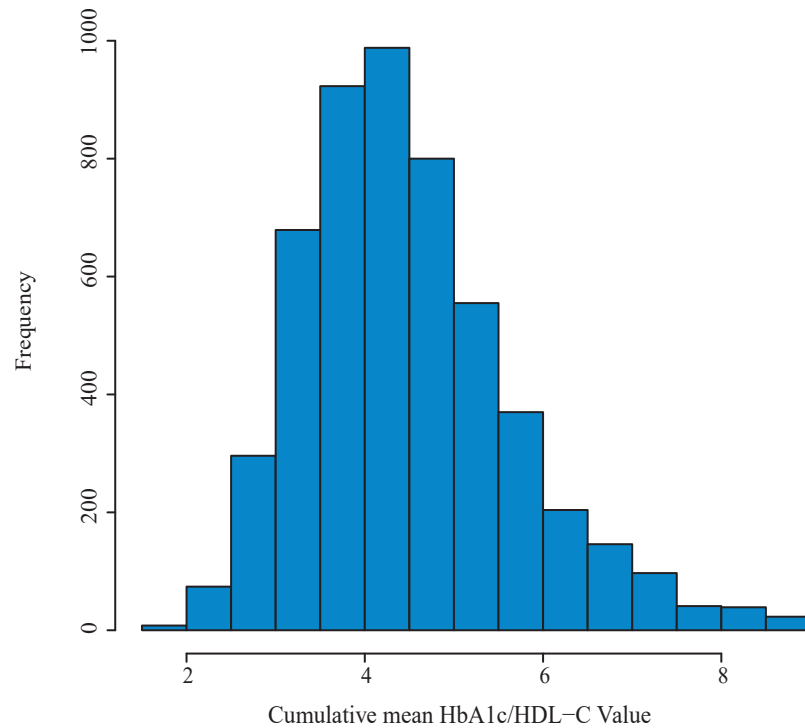

Supplement: Supplementary file 8 — Supplementary Material 8: Supplementary Figure 1 Distribution of the HbA1c/HDL-C in 2011 and cumulative mean HbA1c/HDL-C in 2011-2015. HbA1c, hemoglobin A1c; HDL-C, high-density lipoprotein cholesterol. [file 12944_2025_2438_MOESM8_ESM.pdf]
